# The Dynamic Interplay among Maternal Empathy, Quality of Mother-Adolescent Relationship, and Adolescent Antisocial Behaviors: New Insights from a Six-Wave Longitudinal Multi-Informant Study

**DOI:** 10.1371/journal.pone.0150009

**Published:** 2016-03-18

**Authors:** Elisabetta Crocetti, Silvia Moscatelli, Jolien Van der Graaff, Loes Keijsers, Pol van Lier, Hans M. Koot, Monica Rubini, Wim Meeus, Susan Branje

**Affiliations:** 1 Research Centre Adolescent Development, Utrecht University, Utrecht, the Netherlands; 2 Department of Psychology, University of Bologna, Bologna, Italy; 3 Department of Clinical Developmental Psychology, EMGO Institute for Health and Care Research, VU University Amsterdam, Amsterdam, the Netherlands; 4 Department of Developmental Psychology, Tilburg University, Tilburg, the Netherlands; University of Western Brittany, FRANCE

## Abstract

Adolescents’ behavior is often a matter of concern, given their increased likelihood of enacting antisocial behaviors, which cause disruptions in the social order and are potentially harmful for the adolescents themselves and for the people around them. In this six-wave longitudinal study we sought to examine the interplay among maternal empathy, multiple indicators of mother-adolescent relationship quality (i.e., balanced relatedness, conflict, and support), and adolescent antisocial behaviors rated both by adolescents and their mothers. Participants for the current study were 497 Dutch adolescents (56.9% males) followed from age 13 to 18, and their mothers. A series of cross-lagged panel models revealed reciprocal associations between maternal empathy and mother-adolescent relationship quality and between mother-adolescent relationship quality and adolescent antisocial behaviors. Interestingly, we also found some indirect effects of adolescent antisocial behaviors on maternal empathy mediated by mother-adolescent relationship quality. Overall, this study further highlights a process of reciprocal influences within mother-adolescent dyads.

## Introduction

Adolescent antisocial behavior is often a matter of concern for parents, educators, and for the society at large [1]. Adolescents are more likely than children and adults to engage in antisocial behaviors, such as delinquent and aggressive acts, which cause disruptions in the social order and are potentially harmful for the adolescents themselves and for people around them (for a review see [2]). Thus, it is of utmost importance to understand which factors can reduce adolescent involvement in antisocial behaviors and how these behaviors can impact the relational contexts in which adolescents regularly engage.

A consistent literature has shown that parent-adolescent relationship quality and adolescent antisocial behaviors are strongly related [3–5]. In particular, warm and nurturing relationships are negatively associated with adolescent antisocial behaviors. In order to shed further light on factors influencing adolescent antisocial behaviors it is important to take into account parental characteristics that might explain the degree to which parents are able to maintain a positive relationship quality with their offspring during adolescence. In this respect, parents high in empathy may be better able to understand and to respond to their adolescents’ changing needs. Furthermore, in line with transactional models [6], adolescents might affect with their behaviors quality of parental relationships and even parental characteristics.

Therefore, in this study we sought to study the interplay of maternal empathy, mother-adolescent relationship quality (indicated by high balanced relatedness, high support, and low conflicts; [7–9]), and adolescent antisocial behaviors. We studied this issue in a longitudinal sample consisting of 13 years old adolescents (followed until 18 years old) and their mothers. Doing so, we aimed at shedding further light on the dynamic process by which mothers and adolescents influence each other across adolescence.

### Theoretical Background

#### Mother-Adolescent Relationship Quality and Adolescent Antisocial Behaviors

During adolescence, parent-child relationships undergo substantial changes. Support tends to decline from early to middle adolescence and stabilizes thereafter, while conflict is likely to increase from early to middle adolescence and to stabilize during late adolescence [7, 10–11]. Furthermore, parent-child relationships become more egalitarian during adolescence [7] and levels of balanced relatedness (i.e., the extent to which adolescents feel that parents respect their opinions, wishes, and needs; [9]) tend to increase [12]. These changes are triggered by adolescents’ emerging need for autonomy that requires re-negotiating the relationships with parents, finding a new balance that is appropriate for this developmental phase [13–15].

The extent to which parents and adolescents engage in relationship patterns that are appropriate for this period can affect adolescent psychosocial functioning [16]. In contrast, parental attempts to limit adolescent autonomy are often dysfunctional and result in increasing levels of adolescent problem behaviors [17]. Empirical studies on parent-adolescent relationships and adolescent antisocial behaviors have provided evidence consistent with these considerations. In fact, as highlighted by Hoeve et al.’s [4] meta-analysis, indicators of warm and supportive parent-adolescent relationships are negatively related to adolescent antisocial behaviors, whereas conflicting, rejecting, and overcontrolling parental relationships are positively related to them. Thus, adolescent antisocial behaviors are intertwined with specific indicators of parent-child relationship quality, such as balanced relatedness, support, and conflict.

Importantly, the association between parent-child relationship quality and adolescent antisocial behaviors can be conceptualized as a dynamic process of reciprocal influences [18]. In fact, the view that adolescent behaviors are unilaterally predicted by parents has been progressively substituted by transactional models of development [6, 19–20] that underscore that paternal and child characteristics and outcomes influence each other in a reciprocal way. Consistent with this view, longitudinal studies uncovered reciprocal cross-time associations between parent-adolescent relationship quality and adolescent problem behaviors [5, 21–23]. Doing so, they pointed out that parental influences on children’s development were intertwined with erosion effects, indicating that high levels of various adolescent problem behaviors worsened family relationships over time [12, 24–27].

**Fig 1:**
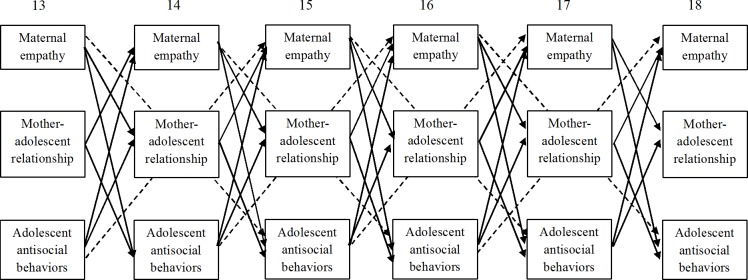
Schematization of the Tested Model. Continuous lines represent main effects and dashed lines represent indirect effects (i.e., effects of maternal empathy on antisocial behaviors mediated by mother-adolescent relationship quality; and effects of antisocial behaviors on maternal empathy mediated by mother-adolescent relationship quality). Stability paths and concurrent correlations were estimated but they are not reported in the figure for sake of clarity.

In line with these considerations, in this study we examined the longitudinal interplay of quality of mother-adolescent relationships and adolescent antisocial behaviors. Specifically, we focused on both concurrent associations and cross-lagged effects. Drawing from the literature reviewed above, we hypothesized to find reciprocal negative associations (at baseline and over time) between indicators of a warm mother-adolescent relationship (high balanced relatedness, high support, and low conflict) and adolescent antisocial behaviors (Fig 1).

#### Maternal Empathy and Mother-Adolescent Relationship Quality

Differences in family relationships might be understood looking at different determinants of parenting [28]. In particular, maternal characteristics may explain the extent to which mothers are able to form warm and supportive mother-adolescent relationships. Recently, a maternal characteristic which is receiving increasing attention is empathy [29].

Empathy is a fundamental social skill, representing the ability to understand and to share the emotional state of another person [30–33]. Mothers with higher levels of empathy might be better able to understand and to respond to the developmental needs of their children [34]. More specifically, maternal empathy may play a role in the quality of the mother-adolescent relationship in several ways. First, the capacity to attune to and to be responsive to children’s feelings is a key element of warm and supportive parenting [34, 35]. Second, a higher tendency to orient to others’ perspectives may facilitate the awareness of adolescents’ need for autonomy and may increase the willingness to support these needs [36]. Third, maternal ability to recognize the adolescents’ feelings and to take their perspective into account when examining a conflict situation should result in fewer conflicts [37]. Previous studies on the association between maternal empathy and parenting in adolescence found that maternal empathy was positively related to maternal support and higher acceptance and promotion of adolescent psychological autonomy [38–39]. In sum, empathy may foster optimal parenting behaviors and therefore enhance a high quality mother-adolescent relationship characterized by high balanced relatedness and support and low conflicts.

Furthermore, the association between maternal empathy and mother-adolescent relationship quality can be conceived as a reciprocal one. In fact, as discussed in the previous paragraph, empathy can improve relationship quality in several ways and, at the same time, a warm mother-adolescent relationship can provide positive feedbacks to mothers, increasing their perception of being able to understand and respond appropriately to adolescents’ needs. So, mother-adolescent relationship quality and maternal empathy can reinforce each other in a positive loop.

Drawing from these considerations, in this study we sought to examine associations between maternal empathy and mother-adolescent relationship quality over the course of adolescence. We expected to find positive reciprocal associations between maternal empathy and indicators of mother-adolescent relationship quality (high balanced relatedness and support, and low conflicts).

#### Maternal Empathy and Adolescent Antisocial Behaviors

Maternal empathy might also affect adolescent antisocial behaviors, both directly and indirectly. For instance, according to the social learning theory [40], maternal empathy could affect adolescent antisocial behaviors directly, since empathic mothers might provide their children with a role model of considerate behavior towards others. In line with this, previous research revealed positive associations between parental empathy and children’s social adjustment [34, 41]. Alternatively, the association between maternal empathy and adolescent antisocial behaviors might be an indirect one and, specifically, be mediated by mother-adolescent relationship quality. In this case, maternal empathy could promote higher mother-adolescent relationship quality which, in turn, should lessen adolescent antisocial behaviors. Recently, Werner et al. [17] provided some evidence of this, showing that maternal empathy decreased adolescent depressive symptoms indirectly through maternal psychological control (while the direct effect was not significant). Thus, maternal empathy might reduce, directly or indirectly, adolescent antisocial behaviors.

Additionally, in line with transactional models of development [6, 19–20], adolescents are likely to influence with their behaviors maternal characteristics, directly or indirectly. A direct effect would imply that adolescent antisocial behaviors negatively affect maternal empathy, since these negative behaviors might make more difficult for parents to be (remain) sensitive to children’s needs as children are damaging their trust. This lower sensitivity might affect the view parents have of their own empathy, as part of their empathy is likely addressed at their own offspring. An indirect effect would involve a mediational process, with the effect of adolescent antisocial behaviors on maternal empathy being mediated by mother-adolescent relationship quality.

Thus, in this study we sought to uncover associations between maternal empathy and adolescent antisocial behaviors. We expected to detect negative reciprocal associations between maternal empathy and adolescent antisocial behaviors. In addressing this hypothesis, we tested both direct and indirect effects to uncover whether maternal empathy has a direct effect on adolescent antisocial behaviors and vice versa (continuous lines represented in Fig 1) or whether these effects are indirect and mediated by mother-adolescent relationship quality (dashed lines in Fig 1).

### The Present Study

Summing up, the purpose of the present study was to disentangle the interplay among maternal empathy, mother-adolescent relationship quality, and adolescent antisocial behaviors. Specifically, we tested in a six-wave longitudinal study involving adolescents (followed from age 13 to 18) and their mothers, reciprocal associations among maternal empathy, mother-adolescent relationship quality (i.e., balanced relatedness, support, and conflict), and adolescent antisocial behaviors (Fig 1). We used a multi-informant design in which indicators of mother-adolescent relationship quality and adolescent antisocial behaviors were rated both by adolescents and their mothers.

## Methods

### Participants

Data were drawn from the ongoing longitudinal RADAR-Y study (Research on Adolescent Development and Relationships–younger cohort). The RADAR study is a population-based prospective cohort study conducted in the Netherlands aimed at examining adolescent development. Because of an interest in both normal and abnormal development, a dual selection procedure was used to oversample adolescents at high-risk for externalizing problem behaviors. In a first step, teacher ratings of behavior problems were collected through the Teacher’s Report Form (TRF [42]). For this purpose, 5,150 early adolescents were assessed when they were 11 or 12 years old. They came from 230 schools located in the Western and central parts of the Netherlands (province of Utrecht and cities of Amsterdam, Rotterdam, The Hague, and Almere). According to the TRF scores, adolescents were assigned to a high-risk or low-risk group. In a second step, early adolescents who fit with the project inclusion criteria (e.g., possibility to include the full family, fluency in the Dutch language reported by each family member, participation of a sibling at least 10 years old) were further selected and invited to participate in the study. The final sample included 497 Dutch families. Detailed information on the overall sampling are reported in van Lier et al. [43].

Participants for the current study were all 497 adolescents (56.9% males; *M*_*age*_ at T1 = 13.03, *SD*_*age*_ = 0.46) and their mothers (*M*_*age*_ at T1 = 44.41, *SD*_*age*_ = 4.45), for a total of 994 respondents. All participating adolescents were attending secondary schools. Most adolescents were native Dutch (95%), lived with both parents (86%), and came from families classified as medium or high socioeconomic status (89%).

Participants provided information for six waves, with one-year intervals between each wave (from 13 to 18 years). Of the original sample, 425 families (86%) were still involved in the study at Wave 6, and the average participation rate over the six waves was 90%. Results of Little’s [44] Missing Completely at Random (MCAR) test yielded a χ^2^/df value of 1.12, suggesting a good fit of sample scores with and without imputation. Therefore, all the 497 mother-adolescent dyads were included in the analyses conducted by means of the Full Information Maximum Likelihood procedure available in M*plus* [45].

### Procedure

The RADAR study has been approved by the Medical Ethical Committee of Utrecht University Medical Centre (the Netherlands). Before the start of the study, adolescents and their parents received written information about the study and they all gave informed consent. Within each year of the study, trained research assistants made appointments for annual home visits. During these visits, participants completed a battery of questionnaires. Research assistants provided verbal instructions in addition to the written instructions that accompanied the questionnaires.

### Measures

#### Maternal Empathy

Mothers reported on their own empathy by completing the Dutch version [46] of the Interpersonal Reactivity Index (IRI; [31]). They filled out 14 items scored on a 5-point scale, ranging from 0 (*doesn’t describe me at all*) to 4 (*describes me very well*). Sample items include: “I often have tender, concerned feelings for people less fortunate than me” and “I try to look at everybody’s side of a disagreement before I make a decision”. In this study, average Cronbach's alpha across six waves was .82 (range: .79-.83).

#### Mother-Adolescent Relationship Quality

Mother-adolescent relationship quality was rated both by the adolescents and their mothers considering multiple indicators (i.e., balanced relatedness, support, and conflict). *Balanced relatedness* refers to the extent to which adolescents feel that their mothers respect their opinions, whishes, and needs. It was rated by both informants with the Balanced relatedness scale ([9]; for the validity of the Dutch version see e.g., [12]). The instrument consists of seven items scored on a four-point scale, ranging from 1 (*absolutely disagree*) to 4 (*absolutely agree*). Sample items include: “My mother respects my decisions”/“I respect my child’s decisions”. In this study, average Cronbach's alphas across six waves were .86 (range: .85-.89) and .88 (range: .85-.90) for adolescent and maternal reports, respectively.

*Support and conflict* were rated by both adolescents and their mothers by completing the respective subscales of the Network of Relationships Inventory (NRI; [8]; for the validity of the Dutch version see [7]). Each participant completed the NRI items using a five-point scale, ranging from 1 (*a little or not at all*) to 5 (*more is not possible*). Sample items include: “How much does your mother really care about you?”/“How much do you really care about your child?” (support, 8 items for each respondent); “How much do you and your mother/child get on each other’s nerves?” (conflict, 6 items). In this study, average Cronbach's alphas across the six waves were and .81 (range: .78-.85) and .93 (range: .90-.95) for the adolescent reports of support and conflict, and .73 (range: .70-.78) and .92 (range: .90-.92) for the maternal reports of support and conflict, respectively.

#### Adolescent Antisocial Behaviors

Both adolescents and their mothers rated adolescent antisocial behaviors. Specifically, adolescents filled out the Dutch version [47] of the antisocial behaviors subscale (30 items) of the Youth Self-Report [42]. Sample items include “I destroy things that belong to others” and “I steal at home”. Mothers completed the Dutch version [48] of the antisocial behaviors subscale (33 items) of the Child Behavior Checklist [49]. Sample items include “My child gets in many fights” and “My child steals outside the home”. All items were scored on a three-point scale (0 = ‘*never’*, 1 = ‘*sometimes’*, and 2 = ‘*often’*). In this study average Cronbach's alphas across six waves were .89 (range: .87-.91) and .91 (range: .89-.92) for adolescent and maternal reports, respectively.

### Strategy of Analysis

Preliminary analyses were conducted in SPSS 22. We computed means and standard deviations for each study variable and bivariate correlations. To address our main research questions, we conducted longitudinal statistical analyses in M*plus* 7.31 [45], using the Maximum Likelihood Robust (MLR) estimator to account for non-normality and non-independence of the data.

Specifically, we tested cross-lagged panel models to examine longitudinal associations between study variables. In each model, we included maternal empathy, one indicator of mother-adolescent relationship quality (i.e., balanced relatedness, support, or conflict), and adolescent antisocial behaviors. In addition, we tested models separately for maternal and adolescent reports of mother-adolescent relationship quality and antisocial behaviors. Therefore, we tested a total of six models in which we examined cross-lagged associations controlling for one-year and two-year stability paths and concurrent associations.

We tested model fit relying on multiple indices [50]: the Comparative Fit Index (CFI) and the Tucker-Lewis Index (TLI), with values higher than .90 indicative of an acceptable fit and values higher than .95 suggesting an excellent fit; and the Root Mean Square Error of Approximation (RMSEA), with values below .08 indicative of an acceptable fit and values less than .05 representing a good fit.

To model the longitudinal associations as parsimoniously as possible, we tested for each model whether cross-lagged effects and T2-T6 correlations were time-invariant. Thus, we compared the model in which these paths were free to vary across time with the constrained model in which they were fixed to be the same. In order to determine significant differences between models at least two out of these three criteria had to be matched: a Satorra and Bentler’s [51] scaled difference chi-square test statistic (Δχ_SB_^2^) significant at *p* < .05, ΔCFI ≥ -.010, and ΔRMSEA ≥ .015 [52–53]. Finally, indirect effects were tested by means of the indirect command procedure available in M*plus* [45].

## Results

### Preliminary Analyses

Means and standard deviations of the study variables are reported in Table 1. Bivariate correlations among study variables at T1 are displayed in Table 2.

**Table 1:** Means (M) and Standard Deviations (SD) for Study Variables.

	T1		T2		T3		T4		T5		T6	
	*M*	*SD*	*M*	*SD*	*M*	*SD*	*M*	*SD*	*M*	*SD*	*M*	*SD*
Maternal empathy (M)	2.75	0.43	2.85	0.42	2.87	0.43	2.87	0.45	2.90	0.45	2.89	0.46
Mother-adolescent relationship quality												
Balanced relatedness (A)	3.27	0.41	3.24	0.44	3.21	0.44	3.20	0.43	3.19	0.44	3.18	0.45
Balanced relatedness (M)	3.27	0.35	3.31	0.36	3.31	0.35	3.35	0.39	3.38	0.38	3.43	0.40
Conflict (A)	1.66	0.58	1.71	0.67	1.75	0.67	1.79	0.69	1.80	0.72	1.74	0.65
Conflict (M)	1.52	0.53	1.55	0.54	1.52	0.50	1.55	0.56	1.50	0.54	1.48	0.54
Support (A)	3.90	0.53	3.81	0.58	3.70	0.60	3.63	0.63	3.65	0.63	3.60	0.64
Support (M)	3.50	0.43	3.44	0.44	3.44	0.44	3.44	0.45	3.41	0.47	3.44	0.50
Adolescent antisocial behaviors (A)	0.35	0.24	0.32	0.27	0.34	0.27	0.35	0.26	0.33	0.25	0.30	0.24
Adolescent antisocial behaviors (M)	0.27	0.24	0.28	0.24	0.26	0.25	0.26	0.26	0.23	0.24	0.20	0.21

*Note*. A = adolescent report; M = maternal report. Response scales: 0–4 (empathy), 1–4 (balanced relatedness), 1–5 (conflict and support), and 0–2 (antisocial behaviors).

**Table 2:** Bivariate Correlations among Study Variables at Time 1.

	1	2	3	4	5
1. Maternal empathy	-	.12**	-.06	.13**	-.04
2. Balanced relatedness	.33***	-	-.33***	.55***	-.21***
3. Conflict	-.10*	-.10*	-	-.33***	.38***
4. Support	.27***	.39***	-.18***	-	-.24***
5. Adolescent antisocial behaviors	-.06	-.12**	.62***	-.17***	-

*Note*. Correlations above the diagonal refers to adolescent (A) reports of mother-adolescent relationship and antisocial behaviors and correlations below the diagonal to maternal (M) reports.

* *p* < .05

** *p* < .01

*** *p* < .001

Bivariate correlations at T2-T6 can be obtained from the first author upon request.

### Cross-Lagged Panel Models

To unravel the longitudinal interplay among maternal empathy, mother-adolescent relationship quality, and adolescent antisocial behaviors, we tested a series of cross-lagged models. First, for each of the six models, we found that the version of the model in which cross-lagged effects and T2-T6 correlations were time-invariant was not substantially different from the version of the model in which these parameters were allowed to vary across time (Table 3). Thus, we could retain the more parsimonious time-invariant models as the final ones.

**Table 3:** Cross-Lagged Models: Fit Indices and Model Comparisons.

	Model fit indices					Model comparison					
Models	χ_SB_^2^	*df*	TLI	CFI	RMSEA [90% CI]	Models	Δχ_SB_^2^	Δdf	*p*	ΔCFI	ΔRMSEA
**Empathy, balanced relatedness, antisocial behaviors (A)**											
M1: Unconstrained	203.549	78	.934	.966	.057 [.047, .067]						
M2: Constrained	252.832	114	.950	.962	.050 [.041, .058]	M2-M1	49.65	36	.065	-.004	-.007
**Empathy, balanced relatedness, antisocial behaviors (M)**											
M1: Unconstrained	270.151	78	.916	.956	.070 [.061, .080]						
M2: Constrained	321.797	114	.938	.953	.061 [.053, .068]	M2-M1	51.178	36	.048	-.003	-.009
**Empathy, conflict, antisocial behaviors (A)**											
M1: Unconstrained	183.206	78	.951	.974	.052 [.042, .062]						
M2: Constrained	243.469	114	.958	.968	.048 [.039, .056]	M2-M1	60.622	36	.006	-.006	-.004
**Empathy, conflict, antisocial behaviors (M)**											
M1: Unconstrained	245.237	78	.935	.966	.066 [.057, .075]						
M2: Constrained	286.841	114	.954	.965	.055 [.047, .063]	M2-M1	47.220	36	.100	-.001	-.011
**Empathy, support, antisocial behaviors (A)**											
M1: Unconstrained	176.224	78	.953	.976	.050 [.040, .060]						
M2: Constrained	230.516	114	.962	.971	.045 [.037, .054]	M2-M1	55.793	36	.019	-.005	-.005
**Empathy, support, antisocial behaviors (M)**											
M1: Unconstrained	242.510	78	.937	.967	.065 [.056, .075]						
M2: Constrained	275.526	114	.958	.968	.053 [.045, .061]	M2-M1	36.964	36	.424	.001	-.012

*Note*. A = adolescent report; M = maternal report. χ^2^ = Chi-Square; df = degrees of freedom; TLI = Tucker-Lewis Index; CFI = Comparative Fit Index; RMSEA = Root Mean Square Error of Approximation and 90% Confidence Interval; Δ = change in parameter. Δχ_SB_^2^ model comparisons are based on Satorra and Bentler’s (2001) scaled difference chi-square test statistic.

**Fig 2:**
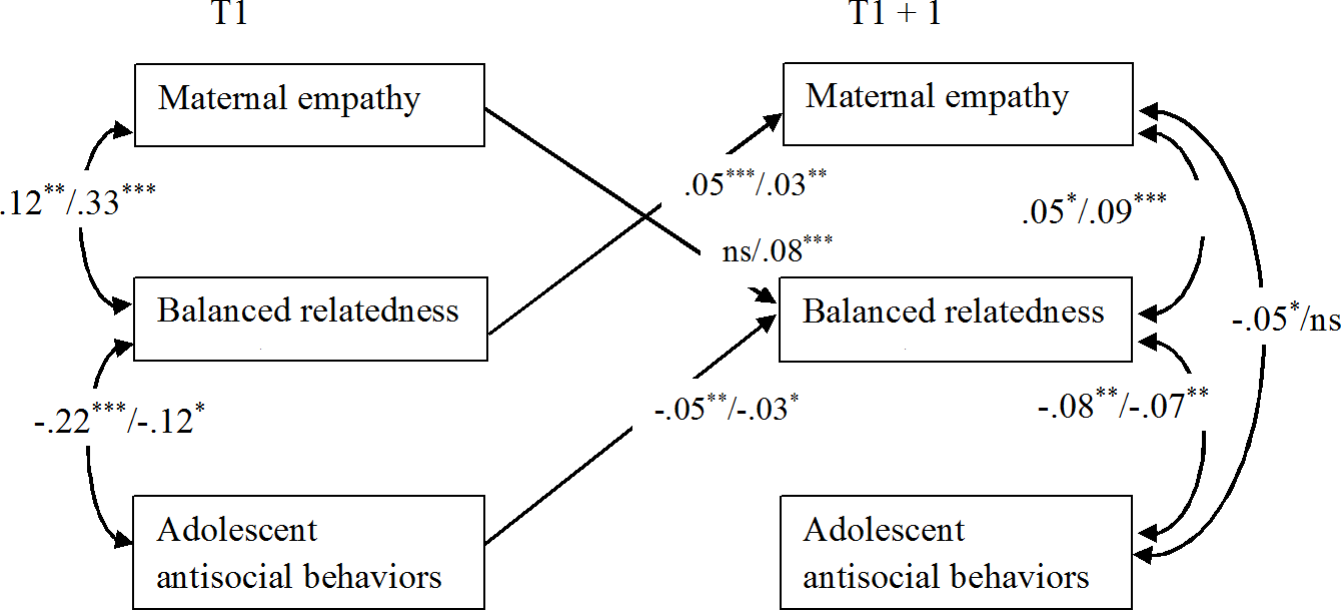
Significant Standardized Results of the Cross-Lagged Models Linking Maternal Empathy, Balanced Relatedness, and Adolescent Antisocial Behaviors (A/M Reports). For each cross-lagged effect and correlation, the first result is based on adolescent reports (A) and the second on maternal reports (M). Since the models with time-invariant coefficients were retained as the final ones, we present only two time points (T and T+1), and all coefficients displayed represent the averaged standardized coefficients over the six time intervals. *p < .05, **p < .01, ***p < .001.

**Fig 3:**
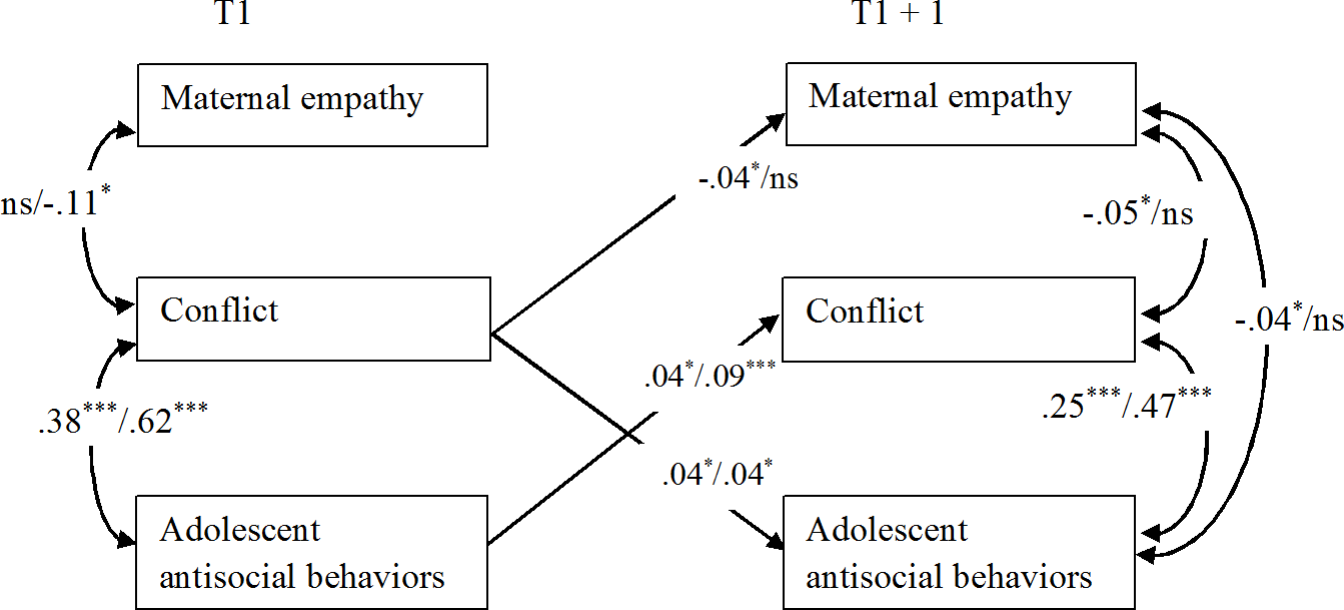
Significant Standardized Results of the Cross-Lagged Models Linking Maternal Empathy, Conflict, and Adolescent Antisocial Behaviors (A/M Reports). For each cross-lagged effect and correlation, the first result is based on adolescent reports (A) and the second on maternal reports (M). Since the models with time-invariant coefficients were retained as the final ones, we present only two time points (T and T+1), and all coefficients displayed represent the averaged standardized coefficients over the six time intervals. *p < .05, **p < .01, ***p < .001.

**Fig 4:**
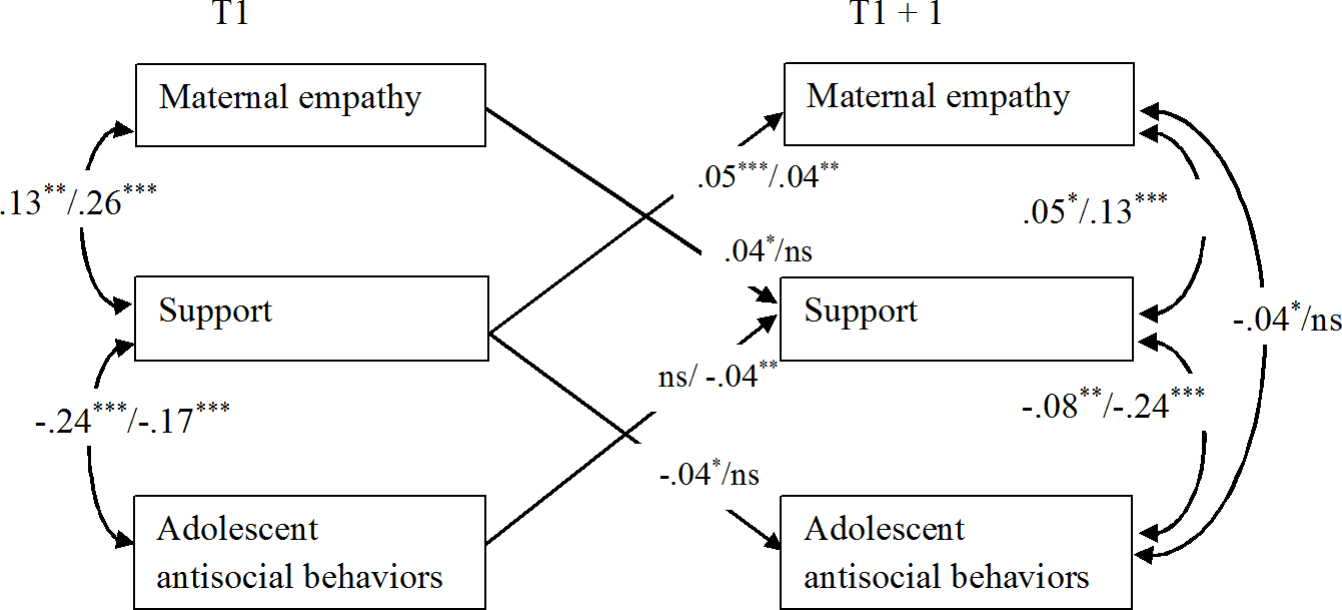
Significant Standardized Results of the Cross-Lagged Models Linking Maternal Empathy, Support, and Adolescent Antisocial Behaviors (A/M Reports). For each cross-lagged effect and correlation, the first result is based on adolescent reports (A) and the second on maternal reports (M). Since the models with time-invariant coefficients were retained as the final ones, we present only two time points (T and T+1), and all coefficients displayed represent the averaged standardized coefficients over the six time intervals. *p < .05, **p < .01, ***p < .001.

Each model fit the data very well (Table 3). One-year and two-year stability paths are reported in Table 4. Results indicated that one-year stability ranged from moderate to large and two-year stability ranged from small to moderate. Significant cross-lagged paths and concurrent correlations are reported in Figs 2–4.

**Table 4:** One-Year and Two-Year Standardized Stability Paths for Study Variables.

	One-year(range)	Two-year (range)
Maternal empathy (M)	.44***–.71***	.30***–.41***
Mother-adolescent relationship quality		
Balanced relatedness (A)	.33***–.46***	.21***–.32***
Balanced relatedness (M)	.30***–.50***	.30***–.36***
Conflict (A)	.38***–.55***	.24***–.35***
Conflict (M)	.43***–.64***	.23***–.29***
Support (A)	.46***–.59***	.18***–.31***
Support (M)	.45***–.63***	.25***–.33***
Adolescent antisocial behaviors (A)	.38***–.71***	.12**–.37***
Adolescent antisocial behaviors (M)	.51***–.78***	.21**–.33***

*Note*. A = adolescent report; M = maternal report.

** *p* < .01

*** *p* < .001

#### Models Linking Maternal Empathy, Balanced Relatedness, and Adolescent Antisocial Behaviors

As shown in Fig 2, concurrent correlations pointed to significant positive associations between maternal empathy and balanced relatedness and negative associations between balanced relatedness and adolescent antisocial behaviors. These findings were very consistent since they were found both at T1 and T2-T6 (i.e., T+1) and for both adolescent and maternal reports. In addition to this, we also found a significant negative T2-T6 correlation between maternal empathy and adolescent antisocial behaviors (with adolescent reports only).

Cross-lagged effects indicated significant reciprocal positive associations between maternal empathy and balanced relatedness (these effects were confirmed by both informants, with only one exception). Furthermore, a significant negative effect of adolescent antisocial behaviors on later balanced relatedness was detected with both adolescent and maternal reports.

#### Models Linking Maternal Empathy, Conflict, and Adolescent Antisocial Behaviors

As displayed in Fig 3, concurrent correlations pointed to significant negative associations between maternal empathy and conflict and positive associations between conflict and adolescent antisocial behaviors. These findings were rather consistent since they were found both at T1 and T2-T6 and most of them were detected with both adolescent and maternal reports. As reported in the prior model, maternal empathy was negatively correlated with adolescent antisocial behaviors (result found at T2-T6 with adolescent reports).

Cross-lagged effects pointed to a significant negative effect of conflict on later maternal empathy for adolescent reports. Furthermore, bidirectional positive effects between conflict and adolescent antisocial behaviors were found for both informants.

#### Models Linking Maternal Empathy, Support, and Adolescent Antisocial Behaviors

As reported in Fig 4, concurrent correlations pointed to significant positive associations between maternal empathy and support and negative associations between support and adolescent antisocial behaviors. These findings were very consistent since they were found both at T1 and T2-T6 and for both adolescent and maternal reports. As already reported in prior models, maternal empathy was negatively correlated with adolescent antisocial behaviors (result found at T2-T6 with adolescent reports).

Cross-lagged effects indicated significant positive reciprocal associations between maternal empathy and support (these effects were confirmed by both informants, with only one exception). Furthermore, we found a significant negative effect of support on later adolescent antisocial behaviors with adolescent reports, while the opposite effects (of adolescent antisocial behaviors on support) was detected with maternal reports.

#### Indirect Effects

We tested indirect effects to examine mediational mechanisms. Findings indicated that mother-effects (i.e., effects of maternal empathy on antisocial behaviors mediated by mother-adolescent relationship quality) were not statistically significant. In contrast, partial support was found for child-effects (effects of adolescent antisocial behaviors on maternal empathy mediated by mother-adolescent relationships quality). More specifically, as reported in Table 5, we found significant indirect effects in two out of six models. In the model with adolescent reports, the negative effect of adolescent antisocial behaviors on maternal empathy was mediated by balanced relatedness; in the model with maternal reports, the negative effect of adolescent antisocial behaviors on maternal empathy was mediated by support.

**Table 5:** Significant Standardized Indirect Effects.

	Indirect effects [95% CI]
**Model with empathy, balanced relatedness, antisocial behaviors (A)**	
Antisocial behaviors T1 > Balanced relatedness T2 > Empathy T3	-.002* [-.005, .000]
Antisocial behaviors T2 > Balanced relatedness T3 > Empathy T4	-.003* [-.005, .000]
Antisocial behaviors T3 > Balanced relatedness T4 > Empathy T5	-.003* [-.005, .000]
Antisocial behaviors T4 > Balanced relatedness T5 > Empathy T6	-.003* [-.005, .000]
**Model with empathy, support, antisocial behaviors (M)**	
Antisocial behaviors T1 > Support T2 > Empathy T3	-.002* [-.003, .000]
Antisocial behaviors T2 > Support T3 > Empathy T4	-.002* [-.003, .000]
Antisocial behaviors T3 > Support T4 > Empathy T5	-.002* [-.003, .000]
Antisocial behaviors T4 > Support T5 > Empathy T6	-.002* [-.003, .000]

*Note*. A = adolescent report; M = maternal report.

* *p* < .05

## Discussion

Adolescence has been traditionally described as a period of turmoil, in which antisocial behaviors reach a peak [54]. In this study, we sought to further improve our understanding of adolescent antisocial behaviors by looking at reciprocal associations between maternal empathy, mother-adolescent relationship quality, and adolescent antisocial behaviors. Results of this six-wave longitudinal study involving adolescents and their mothers highlighted strong linkages between mother-adolescent relationship quality and adolescent antisocial behaviors. Furthermore, findings showed that maternal empathy and mother-adolescent relationship quality were intertwined processes, shedding further light on why some mothers are better able to establish relationship patterns adequate for adolescent developmental needs. Additionally, results also underscored some indirect effects, with the association between adolescent antisocial behaviors and maternal empathy mediated by mother-adolescent relationship quality.

It is worth noting that cross-lagged effects and concurrent T2-T6 correlations were found to be time-invariant, allowing us to model parsimoniously the pattern of associations among study variables over the course of adolescence (13 to 18 years). This suggests that while indicators of relationship quality [7, 11] and rates of antisocial behaviors [55] change during the course of adolescence their reciprocal effects as well as their associations with maternal empathy remain constant. In other words, this highlights that the pattern of reciprocal influences occurring between maternal empathy, mother-adolescent relationship quality, and adolescent antisocial behaviors proceeds with a similar pace over the course of adolescence.

### Disentangling Mother-Adolescent Relationship Quality and Adolescent Antisocial Behaviors

We found very strong support for the association between indicators of mother-adolescent relationship quality and adolescent antisocial behaviors. In fact, both at T1 and at T2-T6, balanced relatedness and support were negatively related, while conflict was positively related, to adolescent antisocial behaviors. Importantly, these findings were very consistent, being detected with both adolescent and maternal reports of mother-adolescent relationship quality and antisocial behaviors. Overall, these results are convergent with a wide cross-sectional literature (for meta-analytic reviews see [4, 56]) showing that parental relationships and adolescent antisocial behaviors are related. Furthermore, our findings are consistent with previous longitudinal evidence highlighting that developmental increases in adolescent problematic behaviors go together with developmental decreases in parent-child relationship quality [5]. Thus, these findings highlight that problematic family relationships and enactment of antisocial behaviors represent two intertwined processes.

In our study, we further disentangled this association by looking at the direction of cross-lagged effects. In the model with *balanced relatedness*, we found that higher levels of antisocial behaviors were related to a decrease in balanced relatedness over time. This result was replicated with both adolescent and maternal reports. It suggests that adolescent antisocial behaviors undermine mothers’ propensity to accept and respect adolescent needs and opinions, since mothers can find their children behaviors unacceptable and harmful. For *conflict*, we found bidirectional associations between conflict and adolescent antisocial behaviors. Thus, conflictual relationships between adolescents and their mothers increase the likelihood that adolescents enact antisocial behaviors, and these behaviors lead to increasing conflicts in a negative loop. Also this set of evidence was confirmed with both adolescent and maternal reports. In contrast, results regarding cross-lagged associations between *support* and adolescent antisocial behaviors differed for adolescent and maternal reports. More specifically, with adolescent reports we found a significant negative effect of support on adolescent antisocial behaviors, while with maternal reports, we found a significant negative effect of adolescent antisocial behaviors on support. This difference seems to point to a sort of “blaming” effect, in which for mothers a relative decrease in support is mainly driven by adolescent negative behaviors while for adolescents it is the other way around. Taken together, results uncovering over-time associations are consistent with transactional models of development [6, 19–20], highlighting a process of reciprocal influences in which a high quality mother-adolescent relationship can limit adolescent antisocial behaviors and adolescent antisocial behaviors can erode family relationships (for a review see [21]).

### Enriching the Picture Looking at Maternal Empathy

In this study, we further expanded our understanding of adolescent antisocial behaviors and mother-adolescent relationship quality by taking into consideration maternal characteristics [28]. In line with the notion that empathy fosters high-quality parent-adolescent relationships [34] and consistent with results of recent studies [17, 29], empathy was found to be related (both at T1 and T2-T6) to indicators of mother-adolescent relationship quality (in particular, balanced relatedness and support), suggesting concurrent developmental processes. These results were largely consistent across adolescent and maternal reports. Importantly, cross-lagged paths clarified the direction of over time influences. It is worth noting that results highlighted that empathy and the quality of mother-adolescent relationship mutually reinforced each other over time, with the strongest effects being from the quality of the relationship to maternal empathy. Interestingly, a high quality mother-adolescent relationship is not only beneficial for adolescents’ empathy as previous studies found [38, 57–58], but also for mothers’ empathy. In fact, our findings indicate that maternal empathy is shaped by the quality of the mother-adolescent relationship and, in a sort of “the rich get richer and the poor get poorer” effect, maternal empathy is reinforced by a mother-adolescent relationship characterized by high balanced relatedness and support whereas it is threatened by a conflictual relationship. Overall, these findings suggest that mothers high in empathy are better able to establish a relationship that is adequate for adolescent increasing autonomy and they further benefit from this, improving their own perception of being empathic.

In this study we also examined direct and indirect effects of maternal empathy on adolescent antisocial behaviors. We found that none of them was significant. Possible explanations for this can be advanced. It could be that maternal empathy model *positive* characteristics and behaviors of the offspring (e.g., adolescents’ own level of empathy [38, 59]). More specifically, it could be that this modeling effect [40] is stronger for characteristics and behaviors with a similar affective quality (e.g., maternal empathy and adolescent prosocial behaviors) and weaker for characteristics and behaviors with a different affective quality (e.g., maternal empathy and adolescent antisocial behaviors). Moreover, it might be that stronger linkages between maternal empathy and adolescent antisocial behaviors emerge when including families in which empathy levels are seriously deficient. This could happen, for instance, in abusing families, in which very low levels of parental empathy go together with child abuse and might result in adolescents showing antisocial behaviors. Future studies are needed to further clarify associations between maternal empathy and adolescent antisocial behaviors in various family contexts.

Regarding effects of adolescent antisocial behaviors on maternal empathy, we did not find direct effects but some support for indirect effects. We found that negative effects of antisocial behaviors on maternal empathy were mediated by balanced relatedness (in the model with adolescent reports) and support (in the model with maternal reports). Taken together, these results indicate that adolescent behaviors lead to a progression erosion in the quality of the mother-adolescent relationships that, in turn, undermine mothers’ capacity of remain empathic and sympathetic with their children. Thus, this evidence points to the active role played by adolescents within the dyadic relationship with the mother [6, 21, 23].

### Strengths and Limitations of this Study and Suggestions for Future Research

This study should be considered both in the light of its strengths and shortcomings, which can suggest venues for future research. A first strength of this study was its fully-recursive longitudinal design. This design allowed us to examine concurrent and over time associations among study variables throughout adolescence. We should note that the cross-lagged effects we found were generally small. However, as clearly explained by Adachi and Willoughby [60], such small sizes in cross-lagged models are still meaningful since controlled for stability paths that account for the majority of variance. An essential venue for future research would be to continue monitoring the interplay of family relationships and antisocial behaviors during and after the transition to emerging adulthood. In particular, it would be very interesting to further unravel these associations in the minority of youth who exhibit life-course-persistent antisocial behaviors as compared to the majority of those for which antisocial behaviors are limited to adolescence [55].

A second strength of this study was the inclusion of both adolescents and their mothers. In this way, we could test if findings were replicated using adolescent and maternal reports of mother-adolescent relationship dimensions and adolescent antisocial behaviors. Overall, results were very consistent across different informants. An important suggestion for future research would be to examine whether these findings could be replicated using observational measures of mother-adolescent interactions.

A further strength of this study was the inclusion of maternal empathy. Doing so, we expanded the focus on the linkages between parental relationships and adolescent antisocial behaviors to examine how this pattern of associations can be further explained taking into account parental characteristics [28]. We found that maternal empathy was related to indicators of mother-adolescent relationship quality whereas it was unrelated, both directly and indirectly, to adolescent antisocial behaviors. Future studies can further enrich this line of research taking into account other parental characteristics, such as parents’ personality, self and identity, and moral reasoning. In this way, it could be possible to further understand which parental characteristics play a leading role in explaining differences in relationships with offspring and in their involvement in antisocial acts.

In addition to this, we should note that a limitation of our study was the solely focus on maternal empathy and the mother-adolescent relationship. Thus, we could not test whether the same results would be replicated considering paternal empathy and indicators of paternal-adolescent relationship quality. Future studies are needed to clarify whether mothers’ and fathers’ effects are comparable.

Finally, our study involved mainly Dutch intact families with medium or high socio-economic status. Thus, we could not deepen to what extent the family social situation could account for some of the effects documented in this study. A main direction for future research could be to disentangle the interplay of parental characteristics, relationship quality, and adolescent antisocial behaviors across different types of families (e.g., divorced families, multi-problematic families, abusing families, and families with low socio-economic status). In this way, it would be possible to further clarify how family and contextual factors (e.g., adolescents coming of age in a condition of poverty) could moderate some effects documented in this study.

### Conclusions

This study provides new insights into our understanding of the interplay among maternal characteristics, mother-adolescent relationship quality, and adolescent antisocial behaviors. In fact, in this multi-wave and multi-informant longitudinal study, we found that indicators of mother-adolescent relationship quality (balanced relatedness, support, and conflict) were reciprocally associated with adolescent antisocial behaviors. Moreover, considering maternal empathy we could gain a better understanding of why some mothers are better able to establish supportive relationships with their offspring and we also found that maternal empathy is influenced (indirectly) by adolescent antisocial behaviors. Overall, this study further highlights the interactive dynamic characterizing the mother-child relationship suggesting that adolescent antisocial behaviors are both fostered by family relationships and, at the same time, modify them, even influencing mothers’ own level of empathy.
